# Exenatide Pretreatment Improved Graft Function in Nonhuman Primate Islet Recipients Compared to Treatment after Transplant Only

**DOI:** 10.1155/2012/382518

**Published:** 2012-09-27

**Authors:** Jill L. Buss, Amer Rajab, Elizabeth D. Essig, Valerie K. Bergdall, Jie Wang, Kwame Osei

**Affiliations:** ^1^Division of Transplantation, Department of Surgery, 1st Floor, The Ohio State University, 395 West 12th Avenue, Columbus, OH 43210, USA; ^2^Division of Endocrinology, Diabetes and Metabolism, 491 McCampbell Hall, 1581 Dodd Drive, Columbus, OH 43210, USA; ^3^Office of Responsible Research Practices, University Laboratory Animal Resources, The Ohio State University, Columbus, 1960 Kenny Road, OH 43210, USA

## Abstract

The GLP-1 receptor agonist, exenatide, has previously been shown to improve insulin secretion, protect beta cells from apoptosis, and promote beta cell regeneration. We propose that pretreatment with exenatide will promote islet graft survival and improve graft function. Pancreatectomized cynomolgus monkeys underwent islet allotransplantation and were treated with exenatide beginning on day 0 or day −2. A third group of animals was treated with an immunosuppressive regimen while a fourth group remained untreated. Fasting blood glucose (FBG) was used to evaluate graft function along with intravenous glucose tolerance tests (IVGTTs) performed at study endpoint (day 10 for untreated and posttransplant exenatide or day 90 for pretreatment exenatide and immunosuppression). The average FBG for pre-treated animals day 5 following transplant was 52.7 ± 14.8 mg/dl, compared to 154.3 ± 105.5 mg/dl for animals treated only following transplant, 59.4 mg/dl ±12.1 for animals treated with immunosuppression, and 265.5 ± 172.3 mg/dl for untreated animals. IVGTTs performed at study endpoint showed normal glucose and insulin curves in the pre-treated exenatide and immunosuppression groups only, with beta cell function actually improving after transplant in the pre-treated group. We conclude, therefore, that exenatide pre-treatment can successfully maintain islet graft survival in nonhuman primates.

## 1. Introduction

Traditionally, type 1 diabetes has been treated by either life-long insulin therapy or, in severe cases, pancreas transplantation. However, frequent episodes of hypoglycemia are common in patients on life-long insulin therapy, and whole pancreas transplantation is an invasive surgical procedure with significant risks. Islet cell transplantation is an attractive alternative to these traditional treatments. However, two of the major limiting factors in the widespread use of islet cell transplantation clinically are the availability of a sufficient number of islets and the inability of current immunosuppressive treatments to protect transplanted islets in the long term.

As of 2010, an estimated 1.5 million people in the United States were diagnosed with insulin-dependent diabetes [1]. According to a 2011 report by the US Department of Health and Human Services, Organ Procurement and Transplantation Network, only 1,562 pancreatic donors were reported [2]. Because islet transplantation is still an experimental procedure, priority of donor pancreata goes to whole organ transplant, thus limiting the potential donors available for islet transplantation. In addition to this already limited availability of pancreata for islet transplantation, most islet transplant recipients require more than one donor in order to acquire a sufficient number of islet cells to achieve insulin independence. This limits the number of people who could be helped by islet cell transplantation even more. Alternative sources of islet cells or development of a method for *β*-cell regeneration is essential to the widespread use of islet transplantation in treating type 1 diabetes.

Despite using 2-3 pancreata for each recipient, however, results reported by the Edmonton group have shown that the rate of success for functional islet grafts in the clinical setting is approximately 80% after 1 year, but only 10% after 5 years [3, 4]. Effects of both auto- and allogeneic immune responses severely limit the long-term success of islet grafts, even when an abundant number of islets have been transplanted. Thus, development of an immunosuppressive treatment strategy that protects islets in the long term is also necessary for the overall success of this procedure.

Many immunosuppressive protocols used in islet cell transplantation to date have relied on calcineurin inhibitors that have been shown to negatively affect pancreatic *β*-cell function and insulin sensitivity. Therefore, despite offering protection from host immune attack, these agents themselves can diminish graft function and contribute to failure of the transplanted islets. Additionally, recipients of islet grafts still suffer the autoimmune effects of diabetes development that led to beta cell destruction initially, thereby affecting function of transplanted islets in the long term.

Incretin mimetics like exenatide are a potentially useful treatment in combination with islet cell transplantation. Exenatide is an analog to glucagon-like peptide 1 (GLP-1) and binds to the GLP-1 receptor on beta cells. GLP-1 and its analogs have been shown to increase insulin production by beta cells in response to glucose [5, 6] as well as promote beta cell regeneration [7, 8], protect from apoptosis [6], and interfere with the autoimmune attack on beta cells [9]. In addition, there is a related class of compounds that inhibits the dipeptidyl-peptidase-IV enzyme that degrades incretin peptides, thus increasing endogenous levels of GLP-1. Pretreatment with one of these compounds has been shown to reduce T-cell migration to islet cells in transplanted nonobese diabetic (NOD) mice [10], raising the question of whether exenatide could have similar effects on immune function.

These far-reaching effects of exenatide on islet cells lead us to believe that exenatide treatment in combination with islet cell transplantation may result in improved long-term islet graft function. Therefore, we propose that pre-treatment with exenatide will improve graft outcomes compared to treatment with more conventional immunosuppression.

## 2. Materials and Methods

### 2.1. Animals

Cynomolgus monkeys (*Macaca fascicularis*), aged 2–4 years, were obtained from Charles River Laboratories (Houston, TX) or Covance Research (Alice, TX). Animals had a starting weight of 4.35 kg ±1.00 (range 2.5–6.1). Animals were housed in individual cages (28 × 30 × 24 in) and given a continuous water supply. Animals were fasted for 12 hours prior to surgery and intravenous glucose tolerance tests (IVGTTs) but were otherwise fed with a regular primate diet supplemented with fresh produce. The procedures described in this study were conducted according to the guidelines set forth in the “Guide for the Care and Use of Laboratory Animals” and by the Institutional Animal Care and Use Committee (IACUC) and University Laboratory Animal Resources (ULAR) [11]. The OSU animal care program is accredited by AAALAC, Int.

### 2.2. Study Design

All animals underwent total pancreatectomies to induce diabetes and were transplanted with islet allografts. The average dose of islets transplanted was 12,110 IEq/kg ±7442.8. Group 1 (*n* = 3) was treated with 5 *μ*g exenatide twice daily (bid) (Amylin Pharmaceuticals, Inc., San Diego, CA) subcutaneously on days −2 to the study endpoint, where day 0 was the day of transplant. Group 2 (*n* = 3) was also treated with exenatide (5 *μ*g bid subq) to study endpoint, but beginning on the day of transplant. Group 3 (*n* = 5) was treated with the following immunosuppression regimen. Induction therapy consisted of rabbit antithymocyte globulin (ATG) (Thymoglobulin, Genzyme, Cambridge, MA) at a dose of 1.5 mg/kg intravenously on days 0–3 and prednisone (Solu-medrol, Pfizer, New York, NY) tapered from 25 to 5 mg over days 0–3. Immunosuppression maintenance consisted of 25 mg cyclosporine (CSA) (Neoral, Novartis, East Hanover, NJ) and 250 mg mycophenolate mofetil (MMF) (CellCept, Roche, Nutley, NJ) given orally from day 0 to the study endpoint. Group 4 (*n* = 4) was untreated and served as the control group.

### 2.3. Surgical Procedures

Outbred pairs of animals were placed under general anesthesia with isoflurane. A midline laparotomy was made in order to perform a total pancreatectomy. The pancreas was dissected from the splenic artery and vein, the portal vein and the duodenum with preservation of the spleen and common bile duct. Blood supply between the spleen and duodenum was double-ligated, and the pancreas removed for islet isolation. Animals were maintained under anesthesia during the islet isolation procedure. Isolated islets from an allogeneic donor were immediately transplanted into the pancreatectomized recipient animal on the same day as pancreatectomy. For Group 1, both donor and recipient animals were pretreated with exenatide for 2 days prior to pancreatectomy and transplantation. Recipients from Groups 2, 3, and 4 received islets that had not been pretreated. The mesenteric vein was located and cannulated with an 18-gauge angiocatheter toward the portal vein. Islets suspended in 50 mL CMRL-1066 transplant media (Mediatech, Herndon, VA) without heparin were slowly injected over 10 minutes. The catheter was removed and the vein ligated. The incision was closed, and animals recovered under close observation, and Buprenex (0.05 mg/kg, Reckitt Benckiser, Berkshire, UK) was given as a postoperative analgesic.

### 2.4. Islet Isolation

Islet isolation was performed according to the modified human islet isolation protocol previously described [12]. Islet number was determined by dithizone staining and conversion to islet equivalents (IEq).

### 2.5. Posttransplant Followup

For the first 48 hours subsequent to transplant, blood glucose was measured twice daily using the tail prick method. A 10 *μ*L drop of blood was drawn from the animal's tail for measurement on a standard glucometer (Ascensia Elite, Bayer Healthcare, Mishawaka, IN). Fasting blood glucose (FBG) was then measured prior to morning feeding once daily through day 7 following transplant and weekly thereafter (for animals that were normoglycemic). Animals with a fasting blood glucose measuring 300 mg/dL or greater were monitored daily and given 2 units of NPH insulin (Eli Lilly, Indianapolis, IN). Intravenous glucose tolerance tests (IVGTTs) were performed prior to pancreatectomy on day 0 and after transplant on days 10 and 90. The study endpoint was considered 90 days after transplant for Groups 1–3 and 10 days after transplant for the untreated Group 4 (due to the poor health noted by day 10 of animals in Group 4). However, two animals in group 1 (exenatide pre-treatment) were monitored beyond 90 days and an additional IVGTT was performed on day 220. Body weights for animals were monitored throughout the study. At the study endpoint, all animals were euthanized following a final IVGTT. Postmortem analysis of the animals' anatomies was performed to visually confirm that no pancreatic tissue remained in the abdomen.

### 2.6. Intravenous Glucose Tolerance Test

Animals were fasted 12 hours prior to each IVGTT. Baseline glucose and insulin were measured at times −5 and 0. A 50% solution of glucose was administered intravenously at time 0. Blood was drawn at times 1, 3, 5, 10, 15, 20, 25, and 30 minutes after glucose administration for measurement of blood glucose and serum insulin levels.

### 2.7. Immunohistochemistry

Upon euthanasia, livers were removed from all animals and preserved in buffered formalin. Paraffin-embedded sections of liver were cut (4 *μ*m) and stained for insulin using a 1 : 100 dilution of guinea-pig polyclonal insulin antibody (Dako North America, Inc., Carpinteria, CA) and for CD3 using a 1 : 400 dilution of rabbit polyclonal CD3 antibody (Dako North America, Inc., Carpinteria, CA). Images were taken at 20x magnification.

### 2.8. *In Vitro* Glucose Stimulation Assay

Freshly isolated islets were handpicked (*n* = 5 islets per replicate × 3 replicates) and incubated at 37°C in either “high” (16.7 mM) or “low” (1.67 mM) glucose for 1 hour. Insulin released in the supernatant was measured using an ELISA kit for human insulin (Dako, Carpinteria, CA). An insulin stimulation index (SI = high/low glucose insulin release) was determined for each sample.

### 2.9. Data Analysis

Area under the curve (AUC) was determined for all IVGTT glucose curves. Glucose disappearance rate constants (k_G_) were used as a measure of glucose tolerance and insulin sensitivity for IVGTTs. k_G_ was calculated as the negative slope of the linear regression for the natural logarithm of glucose from 10 to 30 minutes. Acute insulin response to glucose (AIRg) was used as a measure of first phase insulin secretion during IVGTTs. AIRg was calculated as the mean of insulin at time 3–5 minutes minus the mean at time 0. Beta cell function was measured by the mathematical assessment of glucose regulation called the homeostasis model assessment (HOMA-%B). HOMA-%B was calculated as the average basal insulin (*μ*IU/mL) multiplied by 20 and divided by the average basal glucose (mmol/mL) minus 3.5 [13]. Of note, while HOMA-%B analysis has been validated in clinical use, the model has not been validated in nonhuman primates to date.

### 2.10. Statistical Analysis

All results are expressed as mean ± SEM unless otherwise stated. Student's *t*-test and two-way analysis of variants (ANOVA) were used for parametric analysis. A probability value (*P*) of less than 0.05 is considered statistically significant.

## 3. Results and Discussion

### 3.1. Fasting Blood Glucose

**Figure 1:**
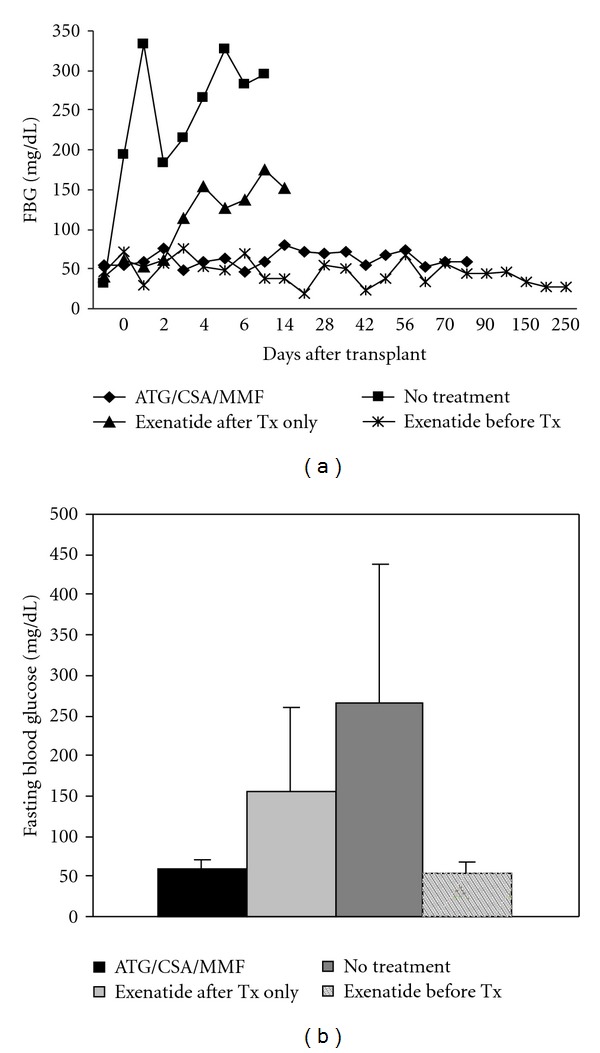
(a) Fasting blood glucose monitoring of transplanted animals. (b) Average fasting blood glucose levels measured at day 5 posttransplant. Untreated animals showed elevated blood glucose levels by day 1 posttransplant, animals treated with exenatide after transplant only showed somewhat elevated blood glucose levels beginning at day 4 posttransplant, while animals treated with ATG/CSA/MMF or pre-treated with exenatide remained normoglycemic throughout the study period. In fact, two animals from the exenatide pre-treatment group remained normoglycemic up to 435 days subsequent to transplant.

Fasting blood glucose levels were used as a determinant for islet graft function posttransplant. Animals in the untreated control group showed elevated blood glucose levels by day 1 posttransplant (Figure 1(a)), with the average FBG at day 5 posttransplant being 265 mg/dL ±172 (Figure 1(b)). Rejection was expected in these animals, and this hyperglycemia was indicative of graft failure. Animals receiving exenatide beginning on the day of transplant had an average FBG at day of 154 mg/dL ±105 (Figure 1(b)). Animals in this group showed variable FBG levels that remained somewhat elevated from day 4 posttransplant to endpoint (Figure 1(a)). While FBG levels were less severely elevated than those of untreated animals and did not consistently meet the criteria for diabetes determination (FBG ≥ 250 mg/dL), they remained elevated throughout the study. Comparatively, ATG/CSA/MMF-treated animals as well as animals pre-treated with exenatide maintained normoglycemia following islet cell transplant (even up to 435 days in two of the exenatide pre-treated animals), indicating functional islet grafts for the duration of the study (Figure 1(a)). On day 5 posttransplant, ATG/CSA/MMF-treated animals had an average FBG of 59.4 mg/dL ±12.1, and animals pre-treated with exenatide had an average day 5 FBG of 52.7 mg/dL ±14.8 (Figure 1(b)). Overall fasting blood glucose was actually significantly lower in animals pre-treated with exenatide compared to animals treated with ATG/CSA/MMF. The immunosuppression strategy employed in this study included the calcineurin inhibitor, cyclosporine, which has been shown to have diabetogenic properties. The use of exenatide in place of this immunosuppression regimen may have reduced the damaging effects of cyclosporine on islet cell function. In addition, these results suggest that exenatide alone is sufficient for the prevention of graft failure.

### 3.2. Intravenous Glucose Tolerance Tests

**Figure 2:**
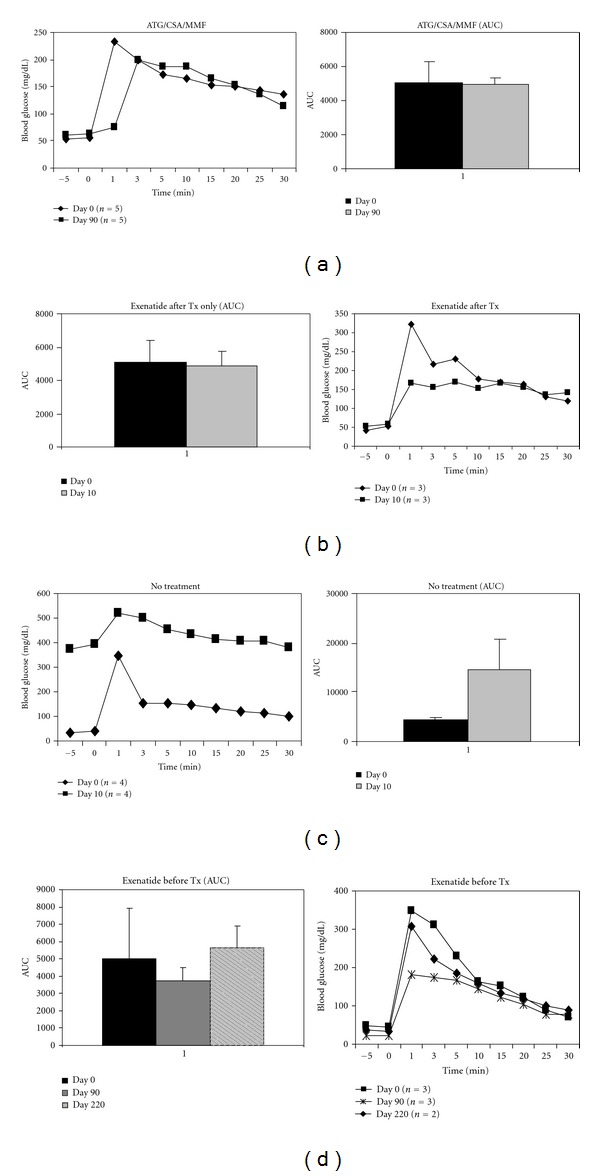
Blood glucose levels measured following intravenous glucose administration (IVGTT) with area under the curve measurements for glucose response for animals treated with ATG/CSA/MMF (a) or exenatide after transplant only (b), untreated (c), and pre-treated with exenatide (d). IVGTTs were performed at baseline prior to pancreatectomy, at day 10 posttransplant for untreated and postexenatide groups, and at day 90 posttransplant for ATG/CSA/MMF and exenatide pre-treatment groups.

**Figure 3:**
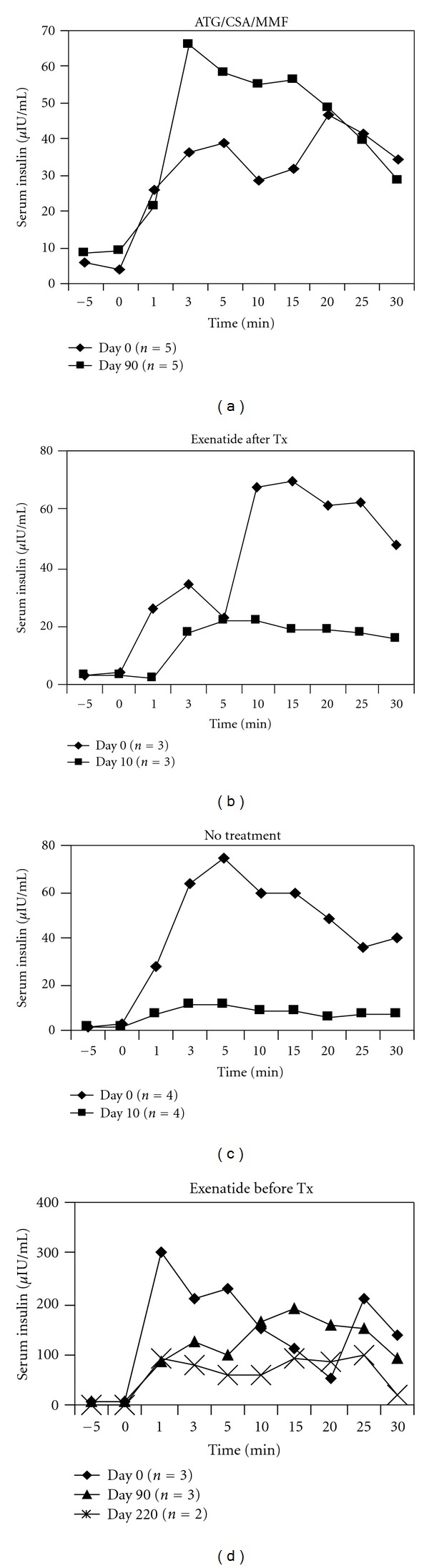
Serum insulin levels measured during IVGTTs as detected by ELISA for animals treated with ATG/CSA/MMF (a) or exenatide after transplant only (b), untreated (c) and pre-treated with exenatide (d).

Results from IVGTTs also indicated functioning islet allografts in animals pre-treated with exenatide or treated with ATG/CSA/MMF, whereas untreated animals had elevated glucose levels and reduced insulin production. Animals receiving exenatide treatment after transplant only also showed impaired insulin production and abnormal glucose curves (Figures 2 and 3). Blood glucose levels were significantly elevated in untreated animals with the AUC being significantly increased following transplant. Animals treated with exenatide following transplant only had an abnormal glucose curve although the AUC was not significantly different (Figure 2). Insulin curves following transplant for both groups were abnormal compared to baseline with no noticeable 2nd phase insulin production and severely reduced insulin levels (Figure 3). On the otherhand, IVGTTs for animals treated with ATG/CSA/MMF as well as animals pre-treated with exenatide were indicative of functional islet grafts, with blood glucose responses resembling that prior to pancreatectomy (Figure 2). There was no significant change subsequent to transplant in the AUC for animals in these groups. Insulin levels actually increased subsequent to transplant in the ATG/CSA/MMF group. For animals pre-treated with exenatide, there was some reduction in insulin over time subsequent to transplant; however, insulin levels were still higher than that of any other treatment group. First and second phase insulin responses also continued to be noted after transplant for both of these groups (Figure 3).

**Figure 4:**
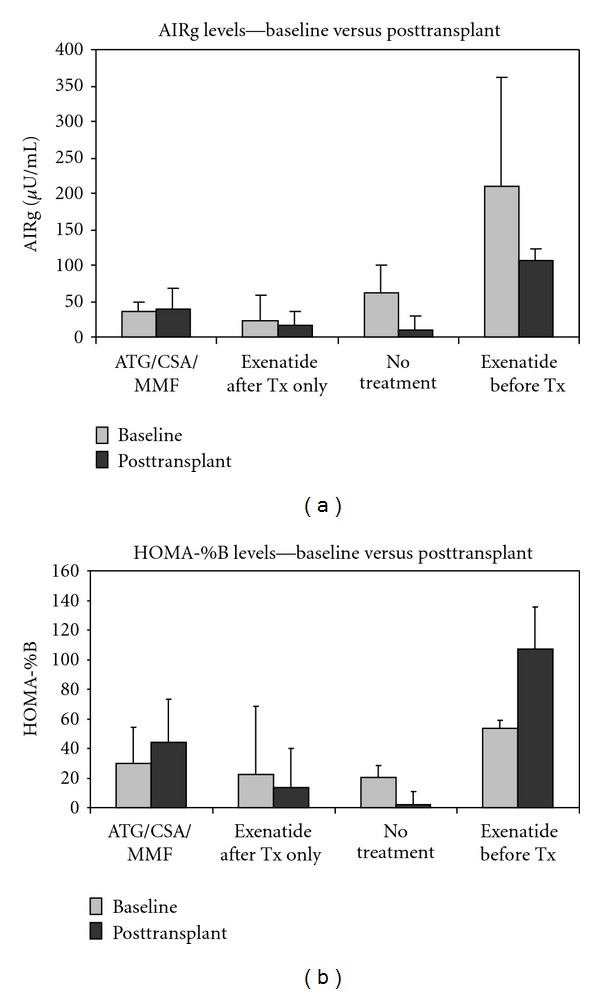
Acute insulin response to glucose (*μ*U/ml) as a measurement of first phase insulin secretion following i.v. glucose administration (a). Beta cell function as determined by homeostasis model assessment (HOMA-%B) (b). Untreated animals and animals treated with exenatide after transplant only were tested at day 0 and at day 10. ATG/CSA/MMF and exenatide pre-treatment groups were tested at day 0 and day 90.

First phase insulin secretion (AIRg) in untreated recipients was severely reduced at 10 days after transplant compared to baseline (9.53 *μ*U/mL ±19.2 versus 61.0 *μ*U/mL ±40.0) (Figure 4(a)). Beta cell function as determined by homeostasis model assessment (HOMA-%B) was also significantly reduced after transplant in untreated animals (1.89% ± 9.14 versus 20.2% ± 8.3 at baseline; *P* = 0.01) (Figure 4(b)). Islet recipients treated with exenatide after transplant only had a mean AIRg at day 0 before pancreatectomy of 23.7 *μ*U/mL ±35.9. At 10 days after transplant, this was reduced to 16.6 *μ*U/mL ±18.1 (Figure 4(a)). Similarly, beta cell function also dropped after transplant from 22.2% ± 46.4 at baseline to 13.8% ± 26.1 (Figure 4(b)). On the other hand, recipients treated with ATG/CSA/MMF maintained a comparable first phase insulin secretion following transplant. AIRg was 35.5 *μ*U/mL ±14.1 at baseline and continued at 39.7 *μ*U/mL ±26.6 ninety days following transplant (Figure 4(a)). Beta cell function increased slightly in this group although the difference was not significant. HOMA-%B in ATG/CSA/MMF-treated animals was 29.9% ± 24.3 at baseline and 43.8%  ± 29.5 at day 90 (Figure 4(b)). Animals that were pre-treated with exenatide showed notably higher first phase insulin secretion at baseline compared to all other groups (211 *μ*U/mL ±150). This did drop after transplant to 105 *μ*U/mL ±19.0 at 90 days but continued to remain significantly higher than other treatment groups, including those treated with immunosuppression (Figure 4(a)). These results suggest that, in addition to protecting islets from rejection, exenatide was able to actually improve graft function relative to regular immunosuppression. However, the specific mechanisms of this have not yet been determined. Baseline beta cell function was also increased in the exenatide pre-treatment group compared to other groups (53.3% ± 5.55), and this actually showed a significant increase after transplant to 107% ± 28.6 at 90 days (Figure 4(b)).

It is known that exenatide increases glucose-dependent insulin secretion. However, in addition to this effect, exenatide has been shown in other studies to promote beta cell regeneration and neogenesis as well. Xu et al. described that treatment of rats with exendin-4 after partial pancreatectomy prevented the development of diabetes with evidence of both replication of preexisting beta cells as well as neogenesis of beta cells from ductal progenitor cells [7]. Another study in streptozotocin-treated rats also indicated an increase in beta cell mass in animals treated with either GLP-1 or exendin-4 compared to those treated only with streptozotocin. This study found that the increased beta cell mass was due primarily to neogenesis from progenitor ductal cells [8].

Additionally, exenatide has been shown to decrease apoptosis of beta cells. *In vitro* analysis of INS-1 cells has shown complete abrogation of apoptosis following pre-treatment with exenatide. This same study also showed a decrease in the oxidative stress-inducing thioredoxin interacting protein (TXNIP) and the apoptotic factors caspase-3 and Bax after *in vivo* treatment with exenatide [6].

A combination of these effects of exenatide on beta cell function, regeneration, and survival likely play a role in the maintenance of islet graft function in the absence of immunosuppression treatment. Further analysis will need to be done, however, to evaluate the roles of these effects on allogeneic graft survival in the nonhuman primate.

### 3.3. Immunohistochemical Analysis of Grafts

**Figure 5:**
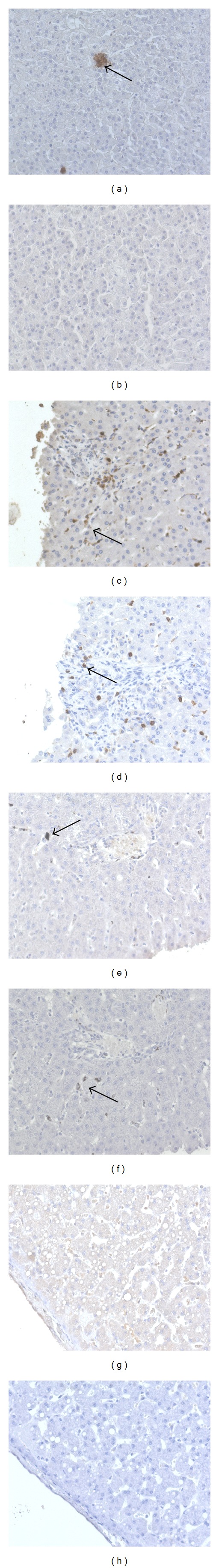
Paraffin-embedded liver sections from islet recipients stained for insulin (a, c, e, g) and CD3 (b, d, f, h), 20x magnification. Animals pre-treated with exenatide showed insulin-positive cells (a) with little evidence of CD3 positive cells (b) (day 435 posttransplant). Animals receiving exenatide after transplant only showed evidence of islet destruction with insulin accumulation in the Kupffer cells of the liver (c). CD3 positivity was also noted throughout the liver in animals treated with exenatide after transplant only (d) (day 10 posttransplant). Minimal evidence for insulin positive islet cells was found in the liver of animals treated with conventional immunosuppression while some residual staining was noted in Kupffer cells of these animals (e). Some CD3 positive cells were also noted in these grafts (f) (day 90 posttransplant). No insulin positivity (g) or CD3 positive cells (h) were found in the livers of untreated animals (day 10 posttransplant).

Liver sections from animals in each group were stained for insulin and CD3 at the conclusion of the study. In animals that were pre-treated with exenatide, islet grafts were evident in the liver as indicated by areas of positive insulin staining. CD3 staining yielded few positive cells in these grafts, suggesting a lack of T-cell infiltration (Figures 5(a) and 5(b)). This correlated with the evidence of graft function indicated by normoglycemia and insulin production in these animals. Comparatively, animals that were treated with exenatide following transplant only showed prominent insulin staining in the Kupffer cells of the liver with almost no evidence of intact islets (Figure 5(c)). This suggested destruction of islet cells in these animals with accumulation of insulin positive regions in the Kupffer cells. CD3 staining also showed diffuse infiltrates of T-cells in these animals (Figure 5(d)). Islet allograft recipients treated with ATG/CSA/MMF showed few insulin-positive islet cells with some accumulation of insulin in Kupffer cells. Additionally, some CD3 positive cells were noted (Figures 5(e) and 5(f)). Although animals in this group maintained normoglycemia, this suggests some immune destruction of these grafts by 90 days subsequent to transplant. Finally, no evidence of islets was found in the livers of untreated animals by insulin staining (Figure 5(g)). This correlates to the hyperglycemia noted in these animals suggesting graft rejection. However, no CD3 positive cells were found by immunohistochemical analysis in these animals either (Figure 5(h)). Hyperglycemia was noted in these animals already at day 1 posttransplant, however, and livers were not collected until day 10 posttransplant, so graft destruction may have already been complete by the time these animals were euthanized.

### 3.4. *In Vitro* Glucose Stimulation and Exenatide Pre-treatment

**Figure 6:**
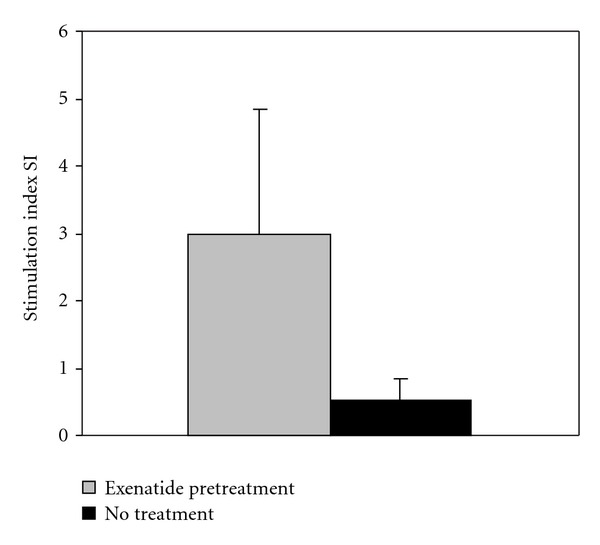
*In vitro* static glucose stimulation assays performed on freshly isolated islets showed a significant improvement in stimulation index of insulin release for islets that had been pre-treated with exenatide *in vivo* compared to untreated islets. *P ≤ 0.05.

Due to our experimental set-up, both donors and recipients in the exenatide pre-treatment group began treatment 2 days prior to transplant. *In vitro* glucose stimulation assays performed on freshly isolated islets in this study indicated a significant improvement in islet function for islets pre-treated with exenatide compared to those that did not receive exenatide treatment. The average stimulation index for islets isolated from animals receiving *in vivo* exenatide pre-treatment was 2.98 ± 1.85. For islets isolated from untreated donor animals (not previously treated with exenatide), the average stimulation index was 0.52 ± 0.32. This was significantly lower than that of exenatide-treated animals (*P* = 0.04) (Figure 6). This suggests that exenatide pre-treated islets were of a higher quality upon transplantation. Improved glucose tolerance, insulin secretion, and beta cell function in animals pre-treated with exenatide support this.

Additionally, even at day 0 prior to transplantation, insulin secretion and glucose tolerance were better in exenatide pre-treated animals compared to other groups. Serum insulin levels produced in response to intravenous glucose administration were also significantly higher in animals pre-treated with exenatide (Figure 2(b)). Because this improvement was noted even at day 0, this suggests that pre-treatment with exenatide may be key in improved graft function and protection over time. Animals treated with exenatide post-transplant only did not show the same improvements and/or maintenance of graft function compared to those receiving pre-treatment. Previous studies have also shown *in vitro* that pre-treatment with exendin-4 improves protection of beta cells from apoptosis [6, 14]. Thus, in addition to showing an increased insulin response to glucose, islets pre-treated with exenatide may have had a higher survival rate following transplant as well. This may also offer some explanation for the improved graft function of exenatide pre-treated animals compared to those treated only with immunosuppression. Therefore, we describe a very unique protocol (*in vitro* and *in vivo*) that has the potential to protect islet cells for transplantation. In a clinical setting, however, cadaveric transplantation would not allow for *in vivo* pre-treatment of islets. This is a limitation to this current protocol and suggests that further study is necessary.

### 3.5. Exenatide Toxicity

There was some initial concern regarding potential side effects of exenatide treatment at this dose in the nonhuman primate. It has been shown previously that exenatide treatment can result in decreased gastric emptying and weight loss. Therefore, animal diet and weight were monitored closely throughout the study. For approximately 2–4 weeks after the start of exenatide treatment, decreased appetite was noted compared to animals not treated with exenatide. This resulted in an average 16.9% weight loss in these animals at day 10 posttransplant. However, after 2–4 weeks of exenatide treatment, the animals' appetites returned to normal, and healthy weight gain was resumed with an average weight gain of 7.59% by day 90. This was compared to an average total weight gain of 10.9% at day 90 in animals not treated with exenatide.

### 3.6. Exenatide and Autoimmunity

*In vitro* studies by other groups have shown that exenatide reduces expression of JAK-1 and STAT-1, which mediate transcriptional effects of interferon-*γ* (INF-*γ*) involved in beta cell apoptosis in the autoimmune development of type 1 diabetes. Microarray analysis and quantitative real-time PCR showed decreases in JAK-1 and STAT-1 expression in both INS-1 cells and isolated human islets after coculture with exenatide [9]. Additionally, another recently published manuscript has also shown that the dipeptidyl peptidase-IV inhibitor, MK0431, which increases circulating levels of incretin hormones like GLP-1, reduced the migration of CD4+ T lymphocytes to transplanted islets. Although autoimmune development of diabetes was not a factor in the model of this study, it is an important factor in the clinical setting of islet cell transplantation and long-term graft survival. The potential of exenatide in blocking this pathway could play a vital role in the long-term success of islet cell transplantation clinically. Whether pre-treatment of potential type 1 diabetic islet cell recipients could promote islet beta cell function and survivability remains to be determined. It can theoretically be argued that exendin-4 could also stunt the cell-mediated and/or humoral (antibody-) mediated islet cell destruction pathways. This remains to be investigated.

## 4. Conclusions

In summary, we have shown in this study that exenatide can improve graft function relative to treatment with immunosuppression and that, in fact, exenatide treatment alone is sufficient in itself in protecting islet allografts from rejection. However, pre-treatment of donors and/or recipients appears to be necessary to achieve this level of graft function. When exenatide treatment of the recipient is not initiated until day 0 and transplanted islets are also untreated, graft function becomes impaired and more closely resembles that of grafts in untreated animals. In addition, this is a pilot study, and the mechanisms of action have not been elucidated. We believe, however, that the roles of exenatide in insulin secretion, beta cell regeneration, and protection from apoptosis are crucial to the success of graft survival. This, along with its potential to protect islets from autoimmune attack, makes exenatide a useful treatment for long-term success in islet transplantation in type 1 diabetic patients. Further studies are needed to test this intriguing hypothesis in type 1 diabetic patients who are candidates for islet cell transplantation.

## Abbreviation

AIRg: Acute insulin response to glucose
ANOVA: Analysis of variance
ATG: Antithymocyte globulin
AUC: Area under the curve
CSA: Cyclosporine
ELISA: Enzyme Linked Immunosorbent Assay
GLP-1: Glucagon-like peptide 1
HOMA-%B: Homeostasis model assessment of beta cell function
IACUC: Institutional Laboratory Animal Care and Use Committee
IEq: Islet equivalent
INF-*γ*: Interferon gamma
IVGTT: Intravenous glucose tolerance test
k_G_: Glucose disappearance rate constant
MMF: Mycophenolate mofetil
NOD: Nonobese diabetic
NPH insulin: Neutral Protamine Hagedorn insulin
SEM: Standard error of measurement
TXNIP: Thioredoxin interacting protein
ULAR: University Laboratory Animal Resources.
